# Potent Bispecific Neutralizing Antibody Targeting Glycoprotein B and the gH/gL/pUL128/130/131 Complex of Human Cytomegalovirus

**DOI:** 10.1128/AAC.02422-20

**Published:** 2021-02-17

**Authors:** Hang Su, Xiaohua Ye, Daniel C. Freed, Leike Li, Zhiqiang Ku, Wei Xiong, Peng Gao, Xinli Liu, Diana Montgomery, Weifeng Xu, Amy S. Espeseth, Dai Wang, Ningning Ma, Tong-Ming Fu, Ningyan Zhang, Zhiqiang An

**Affiliations:** a Wuya College of Innovation, Shenyang Pharmaceutical University, Shenyang, China; b Texas Therapeutics Institute, Brown Foundation Institute of Molecular Medicine, University of Texas Health Science Center at Houston, Houston, Texas, USA; c Merck Research Laboratories, Merck & Co., Inc., Kenilworth, New Jersey, USA; d University of Houston College of Pharmacy, Houston, Texas, USA

**Keywords:** bispecific antibody, monoclonal antibody, neutralizing antibody, human cytomegalovirus, IgG-scFv, gB, pentamer

## Abstract

Human cytomegalovirus (HCMV) is a ubiquitous pathogen that can cause developmental disorders following congenital infection and life-threatening complications among transplant patients. Potent neutralizing monoclonal antibodies (MAbs) are promising drug candidates against HCMV infection. HCMV can infect a broad range of cell types. Therefore, single neutralizing antibodies targeting one HCMV glycoprotein often lack either potency or broad cell-type coverage. We previously characterized two human-derived HCMV neutralizing MAbs. One was the broadly neutralizing MAb 3-25, which targets the antigenic domain 2 of glycoprotein B (gB). The other was the highly potent MAb 2-18, which specifically recognizes the gH/gL/pUL128/130/131 complex (pentamer). To combine the strengths of gB- and pentamer-targeting MAbs, we developed an IgG–single-chain variable fragment (scFv) bispecific antibody by fusing the 2-18 scFv to the heavy-chain C terminus of MAb 3-25. The resulting bispecific antibody showed high-affinity binding to both gB and pentamer. Functionally, the bispecific antibody demonstrated a combined neutralization breadth and potency of the parental MAbs in multiple cell lines and inhibited postinfection viral spreading. Furthermore, the bispecific antibody was easily produced in CHO cells at a yield above 1 g/liter and showed a single-dose pharmacokinetic profile comparable to that of parental MAb 3-25 in rhesus macaques. Importantly, the bispecific antibody retained broadly and potent neutralizing activity after 21 days in circulation. Taken together, our research provides a proof-of-concept study for developing bispecific neutralizing antibody therapies against HCMV infection.

## Abstract (precis)

Human cytomegalovirus (HCMV) is a ubiquitous pathogen that can cause developmental disorders following congenital infection and life-threatening complications among transplant patients. Potent neutralizing monoclonal antibodies (MAbs) are promising drug candidates against HCMV infection.

## INTRODUCTION

Human cytomegalovirus (HCMV), the prototype virus of the betaherpesvirus family, infects up to 85% of the adult population (1). In general, HCMV infection in healthy people causes lifelong asymptomatic latent infection with periodic reactivation and virion shedding (2). An adequate host-derived immune response is critical for maintaining the fine balance of permitting viral reactivation without causing pathogenesis (3). Consequently, HCMV infections are associated with high morbidity and mortality rates in individuals with a weakened immune system, such as AIDS patients, solid-organ transplant recipients, and hematopoietic stem cell transplant recipients (4–7). Congenital HCMV infection is also a leading cause of hearing loss and mental disability in children (8–10). Several antiviral chemotherapies have been approved by the U.S. Food and Drug administration (FDA) and European Medicines Agency (EMA) to treat HCMV infection in immunocompromised patients. Examples include letermovir, ganciclovir, foscarnet, cidofovir, valganciclovir, and fomivirsen. However, the use of these drugs is limited by high toxicity and risk of escape mutations (11, 12). No approved therapy is currently available to prevent or treat congenital HCMV infection (13).

HCMV and other herpesviruses have a multilayered organization. An icosahedral capsid encloses the DNA genome, a tegument layer, and a lipid bilayer envelope decorated with a series of viral glycoproteins (14). Several HCMV glycoproteins or glycoprotein complexes were proven to contribute to HCMV entry into host cells, including gB, gM/gN, gH/gL/gO, and the gH/gL/pUL128/130/131 pentamer (15). HCMV infects *in vivo* a broad variety of cell types, including epithelial cells, endothelial cells, neuronal cells, smooth muscle cells, fibroblasts, monocytes, neutrophils, and hepatocytes (16). The trimeric type III viral fusion protein gB and gH/gL/gO, which binds cellular receptor platelet-derived growth factor receptor α (PDGFR-α), are required for viral entry into all cell types (17–20). The pentamer complex, which interacts with cellular receptors such as NRP-2 and OR14I1, is an additional requirement for HCMV cell-to-cell spread and for HCMV infection of cell types such as epithelial cells, endothelial cells, and leukocytes (21–25). HCMV neutralizing antibodies targeting different viral glycoproteins have been reported. In general, antibodies targeting gB and gH have a broad but moderate HCMV neutralizing potency in most cell types, including epithelial cells, endothelial cells, and fibroblasts. In contrast, pentamer-specific antibodies are extremely potent neutralizers in epithelial and endothelial cells but fail to inhibit HCMV infection of fibroblasts (26, 27). Natural HCMV infection induces the production of potent neutralizing antibodies in humans, which implicates the potential protective effect of virus neutralizing antibodies (28).

Due to the broad cell tropism of HCMV, therapeutic application of single antibodies targeting one viral glycoprotein may not protect all cell types from viral infection and may have the risk of promoting viral escape mutants. A combination of monoclonal antibodies (MAbs) targeting different HCMV glycoproteins can potentially provide a broader protection range and decrease the risk of developing viral resistance (29). An early and robust antibody response targeting the pentamer was associated with a significantly reduced risk of HCMV transmission to the fetus (30). Higher levels of antibody targeting the gB antigenic domain 2 (AD-2), but not the other three gB ADs (AD-1, AD-4, and AD-5), were correlated with decreased incidence of viremia among gB/MF59-vaccinated seropositive solid-organ transplant recipients (31). We previously isolated a panel of neutralizing antibodies from HCMV-positive donors (32). Among them, one gB-specific antibody, MAb 3-25, and one pentamer-specific antibody, MAb 2-18, demonstrated potential for development as anti-HCMV therapeutics. The MAb 3-25 recognizes a totally conserved epitope on gB AD-2 and neutralizes a group of 14 HCMV strains in both ARPE-19 epithelial cells and MRC-5 fibroblast cells, with a 50% inhibitory concentration (IC_50_) of 15 to 188.3 ng/ml (33). MAb 2-18 recognizes a conformational epitope on the pentamer and inhibits HCMV infection of epithelial cells at an IC_50_ of 0.9 ng/ml, but it does not inhibit HCMV infection of fibroblast cells (32, 34). The MAbs 3-25 and 2-18 also inhibited HCMV infection and spread in developing placenta *in vitro* (35). We sought to combine the strengths of broad cell-type coverage by MAb 3-25 and the extremely high potency by MAb 2-18. To this end, we developed and evaluated a MAb 3-25- and MAb 2-18-based tetravalent IgG–single-chain variable fragment (scFv) bispecific HCMV neutralizing antibody as a drug candidate for HCMV infection.

## RESULTS

### Characterization of the scFv fragment of MAbs 2-18 and 3-25.

**FIG 1:**
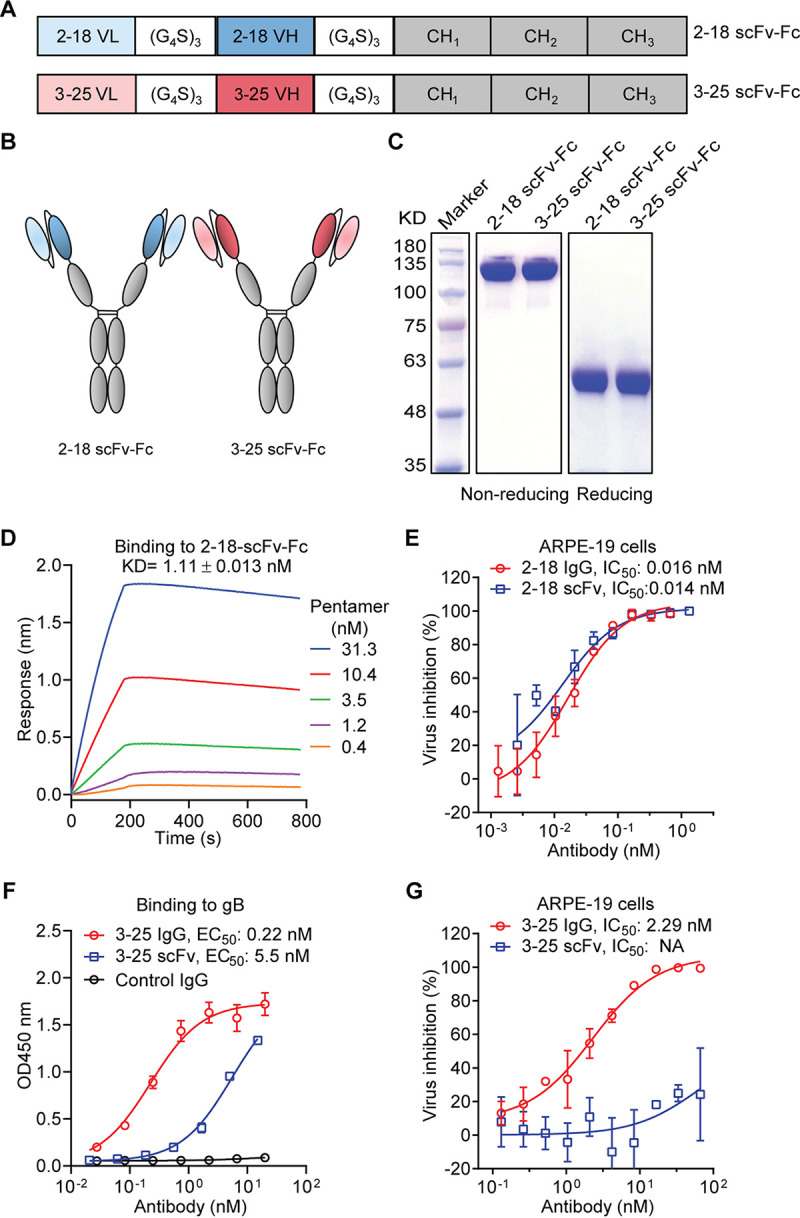
Characterization of scFv fragments of MAbs 2-18 and 3-25. (A and B) Schematic diagrams of scFv-Fc constructs of MAbs 2-18 and 3-25. (C) Purity of the 2-18 scFv-Fc and 3-25 scFv-Fc and parental MAbs was evaluated by SDS-PAGE and Coomassie blue staining under nonreducing and reducing conditions. (D) Interaction between 2-18 scFv-Fc and soluble pentamer was determined via a biolayer interferometry (BLI) assay. (E) *In vitro* HCMV neutralization activities of MAb 2-18 in scFv-Fc format and IgG format in ARPE-19 cells. (F) Binding of MAb 3-25 in scFv-Fc format and IgG format to recombinant gB protein as determined by ELISA. A dengue virus-specific human IgG1 antibody served as a negative control. (G) *In vitro* HCMV neutralization activities of MAb 3-25 in scFv-Fc or IgG format in ARPE-19 cells. The bars indicate mean ± SD values from replicate wells. The IC_50_ and EC_50_ were calculated by nonlinear fit of virus inhibition percentage or OD_450_ readings versus antibody concentrations using GraphPad Prism 5 software.

We aimed to build a bispecific antibody combining the broad cell-type coverage of the gB-specific MAb 3-25 and the high potency of the pentamer-specific MAb 2-18. We previously demonstrated that bivalency of MAb 3-25 is required for HCMV neutralization (33). Thus, we chose a tetravalent IgG-scFv as the bispecific antibody format that preserves bivalency of MAb 3-25. First, we evaluated the functionality of MAbs 3-25 and 2-18 in the scFv format. The variable light-chain (VL) fragment and variable heavy-chain (VH) fragments of MAb 3-25 or 2-18 were joined with a (G_4_S)_3_ linker and then fused at the N terminal of human CH1-Fc, which results in two proteins termed 2-18 scFv-Fc and 3-25 scFv-Fc, respectively (Fig. 1A and B). After transient expression in cells of the human embryonic kidney line HEK293, the 2-18 scFv-Fc and 3-25 scFv-Fc proteins were purified with protein A Sepharose resin. As expected, SDS-PAGE and Coomassie blue staining showed purified 2-18 scFv-Fc and 3-25 scFv-Fc generated higher bands under the nonreducing condition and lower bands under the reducing condition (Fig. 1C). The protein A sensor captured 2-18 scFv-Fc bound to soluble pentamer with nanomolar binding affinity (1.11 ± 0.013 nM), as assessed by a biolayer interferometry (BLI) assay (Fig. 1D). The 2-18 scFv-Fc protein was able to neutralize HCMV infection of ARPE-19 cells with the same potency as the 2-18 IgG (Fig. 1E). The gB binding 50% effective concentration (EC_50_) of 3-25 scFv-Fc was about 25-fold lower than that of 3-25 IgG by enzyme-linked immunosorbent assay (ELISA) (Fig. 1F), indicating a substantially reduced gB binding activity of 3-25 scFv-Fc. In addition, the 3-25 scFv-Fc lost HCMV neutralizing activity in ARPE-19 cells, while the 3-25 IgG neutralized HCMV infection at an IC_50_ of 2.29 nM (Fig. 1G). These results showed that the scFv format of MAb 2-18 but not MAb 3-25 retained comparable binding and neutralizing activities to the parental antibody.

### Design, construction, and dual specificity of the HCMV-bispecific antibodies.

**FIG 2:**
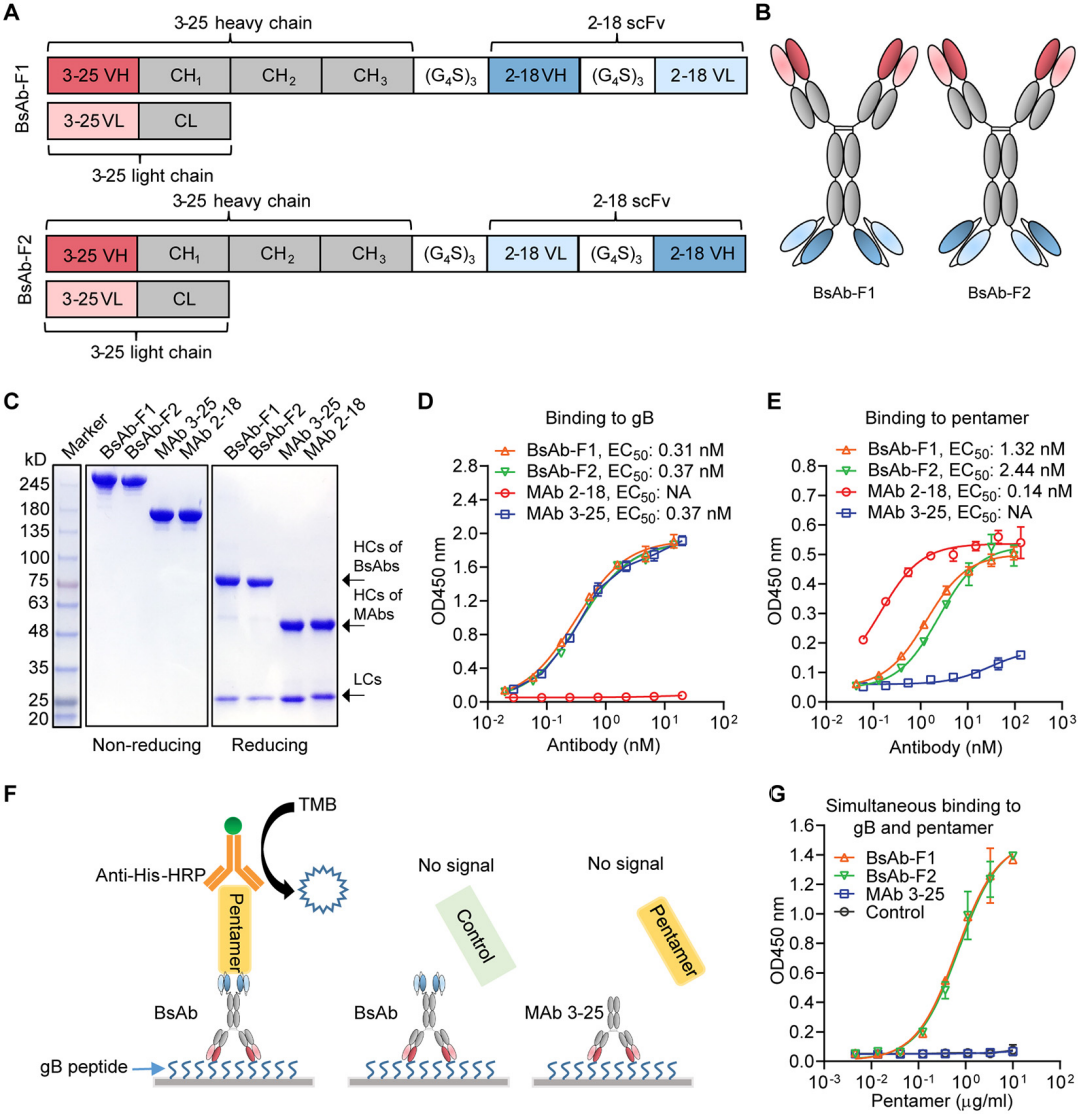
Design, construction, and characterization of HCMV-bispecific antibodies. (A and B) Schematic diagrams of the two tetravalent IgG-scFv bispecific antibodies BsAb-F1 and BsAb-F2. The 2-18 scFv was connected to the C terminus of MAb 3-25 HC via a (G_4_S)_3_ linker. BsAb-F1 has a VH-VL arrangement of 2-18 scFv; BsAb-F2 has a VL-VH arrangement of 2-18 scFv. (C) Purity of the bispecific IgG-scFv and parental MAbs was detected by SDS-PAGE and Coomassie blue staining under nonreducing and reducing conditions, respectively. (D and E) The binding of bispecific IgG-scFv to gB (D) and pentamer (E) was measured by ELISA and compared to that of the parental antibodies. Binding of the antibodies was detected using anti-human Fc antibody conjugated with HRP. (F) A diagram illustrating the sandwich ELISA used to determine the simultaneous binding of bispecific antibodies to gB peptide and pentamer. A 15-mer gB peptide containing the MAb 3-25 epitope was coated on the plate and incubated with 2 μg/ml of bispecific antibodies or MAb 3-25, followed by incubation with titrated recombinant pentamer or a control protein. An HRP-conjugated anti-His antibody was used to detect the binding of pentamer or control protein that has a 6×His tag. (G) Simultaneous binding of bispecific antibodies to gB peptide and pentamer was determined as illustrated in panel F. The bars indicate mean ± SD OD_450_ readings from replicate wells.

Based on the characterizations of the scFv format of MAbs 3-25 and 2-18, we built the tetravalent IgG-scFv bispecific antibody with MAb 2-18 as the scFv component and MAb 3-25 as the IgG component. For construction of IgG-scFv, the original light chain (LC) of MAb 3-25 was used. The heavy chain (HC) of MAb 3-25 was modified by connecting it to the 2-18 scFv at the C terminus via a (G_4_S)_3_ linker. Two versions of tetravalent IgG-scFv bispecific antibodies were designed by shuffling the positions of VH and VL of 2-18 scFv. The bispecific antibody construct 1 (BsAb-F1) has a VH-VL orientation, and BsAb-F2 has a VL-VH orientation (Fig. 2A and B). Plasmids encoding the modified HC and original LC of MAb 3-25 were confirmed by sequencing and cotransfected into HEK293 cells for expression. The bispecific antibodies secreted in cell culture medium were purified using protein A Sepharose resin and analyzed using SDS-PAGE and a Coomassie blue staining assay. Under the nonreducing condition, BsAb-F1 and BsAb-F2 both showed a dominant band of ∼245 kDa, in contrast to and above the∼150-kDa band produced by the parental MAbs 3-25 and 2-18 (Fig. 2C). Under the reducing condition, both BsAb-F1 and BsAb-F2 produced two major bands: an extra-long heavy chain (∼75 kDa) and a normal light chain (∼25 kDa) (Fig. 2C). These results suggest that the bispecific antibodies were well assembled in the HEK293 expression system. In addition, the different VH/VL orientations of the 2-18 scFv did not impact the expression of the bispecific antibodies as the yields of BsAb-F1 and BsAb-F2 were comparable by transient expression.

Binding of the bispecific antibodies on a plate coated with soluble gB and pentamer was evaluated by ELISA. As expected, the parental antibodies bound either pentamer or gB, while BsAb-F1 and BsAb-F2 bound both gB and pentamer (Fig. 2D and E). The binding EC_50_ values of BsAb-F1, BsAb-F2, and MAb 3-25 to gB were 0.31, 0.37, and 0.37 nM, respectively (Fig. 2D), indicating a comparable gB binding activity of the bispecific antibodies and MAb 3-25. The binding EC_50_ values of BsAb-F1, BsAb-F2, and MAb 2-18 to pentamer were 1.32, 2.44, and 0.14 nM, respectively (Fig. 2D). The pentamer binding EC_50_ values of the bispecific antibodies were 9 to 14 times weaker than that of MAb 2-18 (Fig. 2D and E), suggesting a compromised pentamer binding ability of the bispecific antibodies. Simultaneous binding of the bispecific antibodies to pentamer and a gB peptide that contains the MAb 3-25 epitope was evaluated by a sandwich ELISA. As illustrated in Fig. 2F, the bispecific antibodies were captured by the gB peptide applied as a coating on a 96-well plate and then incubated with soluble pentamer containing a 6×His tag. Binding of pentamer was detected by a horseradish peroxidase (HRP)-conjugated anti-His tag antibody. MAb 3-25 and a control protein with a 6×His tag were included as negative controls. As expected, binding of pentamer was detected when BsAb-F1 and BsAb-F2 were added but not when MAb 3-25 was added (Fig. 2G). In addition, no binding of the His-tagged control protein to bispecific antibodies was detected (Fig. 2G). These results demonstrate that the BsAb-F1 and BsAb-F2 have dual binding activity to gB and pentamer.

**FIG 3:**
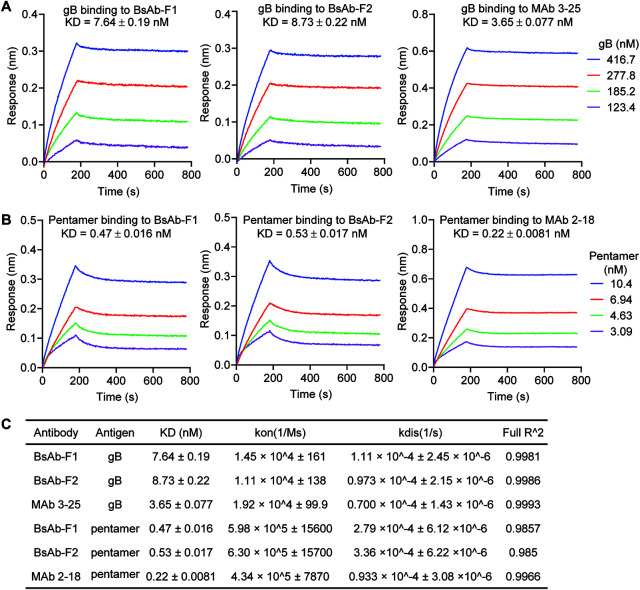
The binding of the bispecific antibodies to gB and pentamer by a BLI assay. Protein A biosensors were used to capture the indicated antibodies. The biosensors loaded with antibodies were then dipped in titrated (A) gB or (B) pentamer solutions. One loaded biosensor was dipped in buffer-only solution to serve as a reference. The data were reference subtracted and then fitted to a 1:1 binding model using the Octet Data Analysis software. (C) The kinetic constants and fitting parameters for gB and pentamer binding to the antibodies.

The binding of gB and pentamer to the bispecific or parental antibodies that were captured on protein A biosensors was assessed by a BLI assay. Consistent with our ELISA results, the captured BsAb-F1 and BsAb-F2 interacted with both gB and pentamer in solution (Fig. 3A). The apparent binding affinities of soluble gB to captured BsAb-F1, BsAb-F2, and MAb 3-25 were 7.64, 8.73, and 3.65 nM, respectively (Fig. 3A and C). The binding affinities of soluble pentamer to captured BsAb-F1, BsAb-F2, and MAb 2-18 were 0.47, 0.53, and 0.20 nM, respectively (Fig. 3B and C). The association (*k*_on_) and dissociation (*k*_dis_) rates measured are listed in Fig. 3C. Interestingly, the binding affinities of gB and pentamer to the captured bispecific antibodies by BLI assay were about twice that of the parental antibodies (Fig. 3C), suggesting that the binding activities of bispecific antibodies were only slightly weaker than that of the parental antibodies. These results demonstrate that BsAb-F1 and BsAb-F2 have dual binding specificities and high binding affinity for gB and pentamer, with BsAb-F1 showing a marginally stronger binding than BsAb-F2.

### Functional characterization of the HCMV-bispecific antibodies *in vitro*.

**FIG 4:**
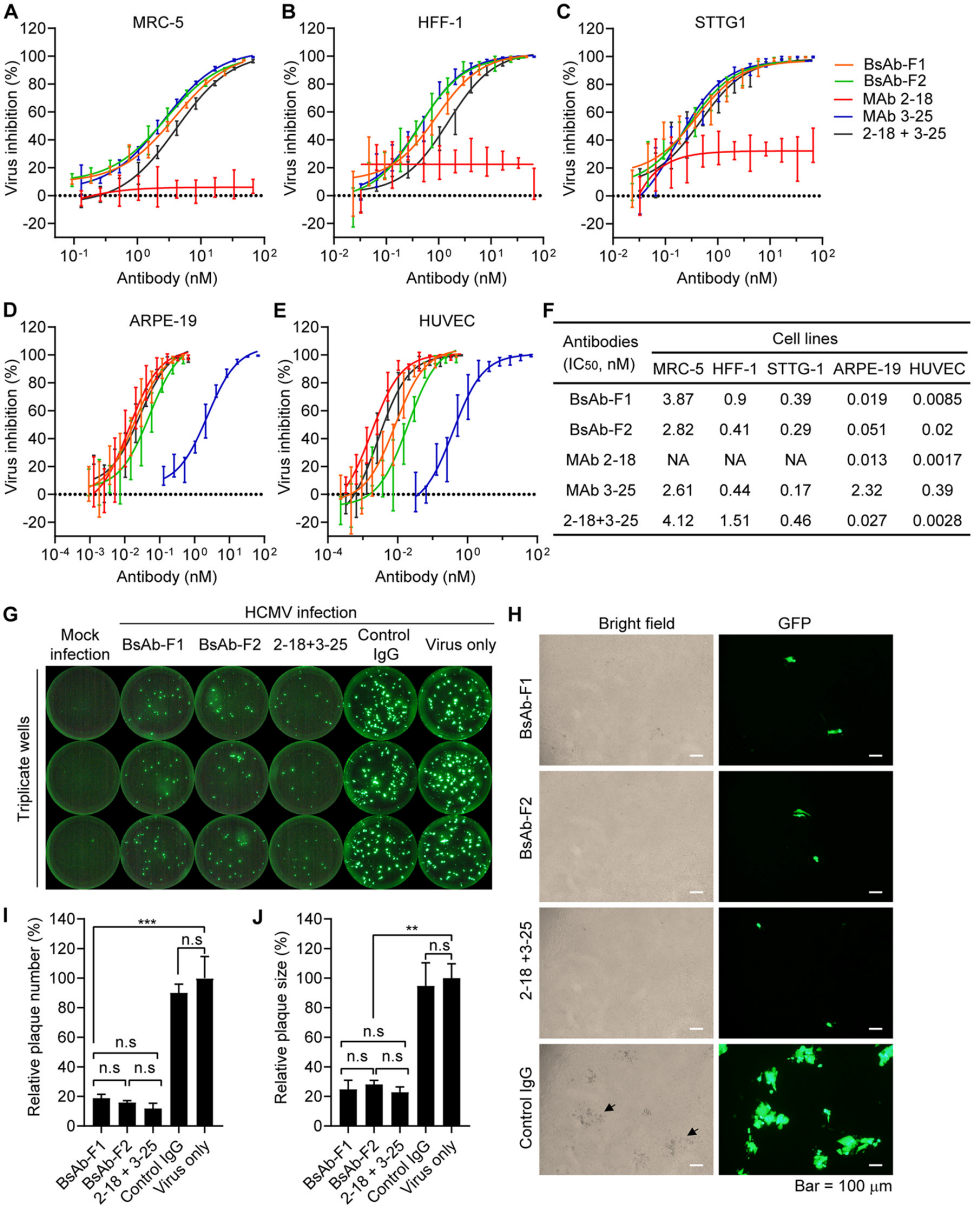
Functional characterization of HCMV-bispecfic IgG-scFv antibodies *in vitro*. (A to F) The neutralization efficacies of indicated antibodies against HCMV strain AD169rev-GFP in (A) MRC-5 cells, (B) HFF-1 cells, (C) STTG1 cells, (D) ARPE-19 cells, and (E) HUVECs. (F) The IC_50_s were calculated by nonlinear fit of virus inhibition percentage versus concentration (nM) using GraphPad Prism 5 software. Data are representative of two independent experiments with four replicate wells for each concentration. (G and H) Inhibition of postinfection viral spreading. Confluent ARPE-19 cells grown in a 96-well plate were infected with AD169rev-GFP. At 3 days postinfection, the virus-containing culture medium was replaced with fresh medium containing 10 μg/ml of the indicated antibodies, and the cells were further cultured. Mock-infected and virus-only wells served as controls. Whole-well GFP images were captured using a CTL Immunospot analyzer at 14 days postinfection. (G) Representative whole-well images for GFP expression. (H) Representative pictures showing the GFP plaques and the cytopathic effects caused by HCMV infection of the same area. Pictures were acquired by an Olympus fluorescence microscope at 14 days postinfection. Size bars = 100 μm. The black arrows indicate examples of cytopathic effects. (I) The number of GFP plaques and (J) the sizes of the GFP plaques in single wells as shown in panel G were quantified by ImageJ software. Data are shown as relative percentages of the number or size of GFP plaques in antibody-treated wells to those of virus-only controls. Statistical significance was determined by unpaired two-tailed Student's *t* test. n.s., not significant (*P* > 0.05); **, *P* < 0.01; ***, *P* < 0.001. The error bars indicate mean ± SD values from replicate wells.

Having demonstrated that the IgG-scFv bispecific antibodies have dual binding specificities to HCMV gB and pentamer, we next assessed whether the bispecific antibodies have combined neutralizing activities of their parental antibodies in four different types of human cells: fibroblast cells (MRC-5 and human foreskin fibroblast-1 [HFF-1]), neuronal cells (STTG1), adult retinal pigment epithelial cells (ARPE-19), and umbilical vein endothelial cells (HUVECs). The AD169-based HCMV strain AD169rev-GFP was used in the neutralization assay. This strain was modified for restoration of pentamer expression and insertion of an in-genome green fluorescent protein (GFP) expression cassette (36). Two single MAbs and a combination of MAbs 3-25 and 2-18 (here “3-25 + 2-18”) were included in the neutralization activity assessment. As shown in Fig. 4A to F, the gB-specific MAb 3-25 inhibited virus infection of all four types of cells, with IC_50_ values of 0.17 to 2.61 nM. The pentamer-specific MAb 2-18 inhibited virus infection in ARPE-19 epithelial cells and HUVECs, with IC_50_s of 0.013 and 0.0017 nM, respectively. As expected, MAb 2-18 did not inhibit virus infection of fibroblast cells (MRC-5 and HFF-1) or human neuronal cells (STTG1) (Fig. 4A and C to F). However, the virus-inhibiting activities of MAb 2-18 in epithelial cells (ARPE-19) and endothelial cells (HUVECs) were about 177 times and 228 times more potent than those of MAb 3-25, respectively (Fig. 4D to F). Importantly, both BsAb-F1 and BsAb-F2 not only inhibited virus infection of fibroblast cells (MRC-5 and HFF-1) and human neuronal cells (STTG1), with comparable IC_50_ values to those of MAb 3-25 and the 3-25 + 2-18 combination (Fig. 4A to F), but also inhibited virus infection of epithelial cells (ARPE-19) and endothelial cells (HUVECs), with comparable IC_50_ values to those of MAb 2-18 and the 3-25 + 2-18 combination (Fig. 4A to F). These results demonstrate that the bispecific antibodies have the combined neutralization potency and expanded cell-type coverage of the parental antibodies.

The viral fusion protein gB and the pentamer complex were shown to be essential for cell-to-cell spread of HCMV (20, 37). We previously showed that the gB-specific MAb 3-25 can inhibit postinfection cell-to-cell spreading (33). We performed the same experiment to determine whether the bispecific antibodies can also inhibit postinfection viral spreading. Confluent ARPE-19 cells grown in a 96-well plate were infected with AD169rev-GFP for 3 days. The virus-containing medium was then replaced with antibody-containing medium, and the cells were incubated at 37°C with 5% CO_2_. Virus infection was examined at 14 days postinfection through GFP expression. Whole-well images were acquired using a CTL Immunospot analyzer. As shown in Fig. 4G, bright GFP plaques were only visible in the virus-infected wells. More GFP plaques were detected in the virus-only and control IgG wells than in the wells with the bispecific antibodies and the 2-18 + 3-25 combination. Under a fluorescence microscope, GFP plaques of cells treated with the HCMV-bispecific antibodies and the 2-18 + 3-25 combination were smaller than those treated with control IgG, and the presence of GFP plaques was also consistent with the cytopathic effects caused by virus infection, as observed under bright-field microscopy (Fig. 4H). The total number and average size of GFP plaques in single wells were quantified using Image J software. The number of GFP plaques in wells with the BsAb-F1 and BsAb-F2 were about 18.7 and 15.8% of those in the virus-only control, respectively (Fig. 4I), indicating that the bispecific antibodies potently inhibited free virus release of infected cells. The sizes of GFP plaques in wells with BsAb-F1 and BsAb-F2 were about 24.8 and 28.2% of those in the virus-only control, respectively (Fig. 4J), suggesting that the bispecific antibodies were capable of inhibiting viral cell-to-cell spreading postinfection. Importantly, the numbers and sizes of GFP plaques in wells with the bispecific antibodies and the 2-18 + 3-25 combination have no significant difference (Fig. 4I and J). These results demonstrate that the IgG-scFv bispecific antibodies efficiently inhibited postinfection virus release and cell-to-cell spread.

### Pharmacokinetics of the HCMV-bispecific antibody in rhesus macaques.

**FIG 5:**
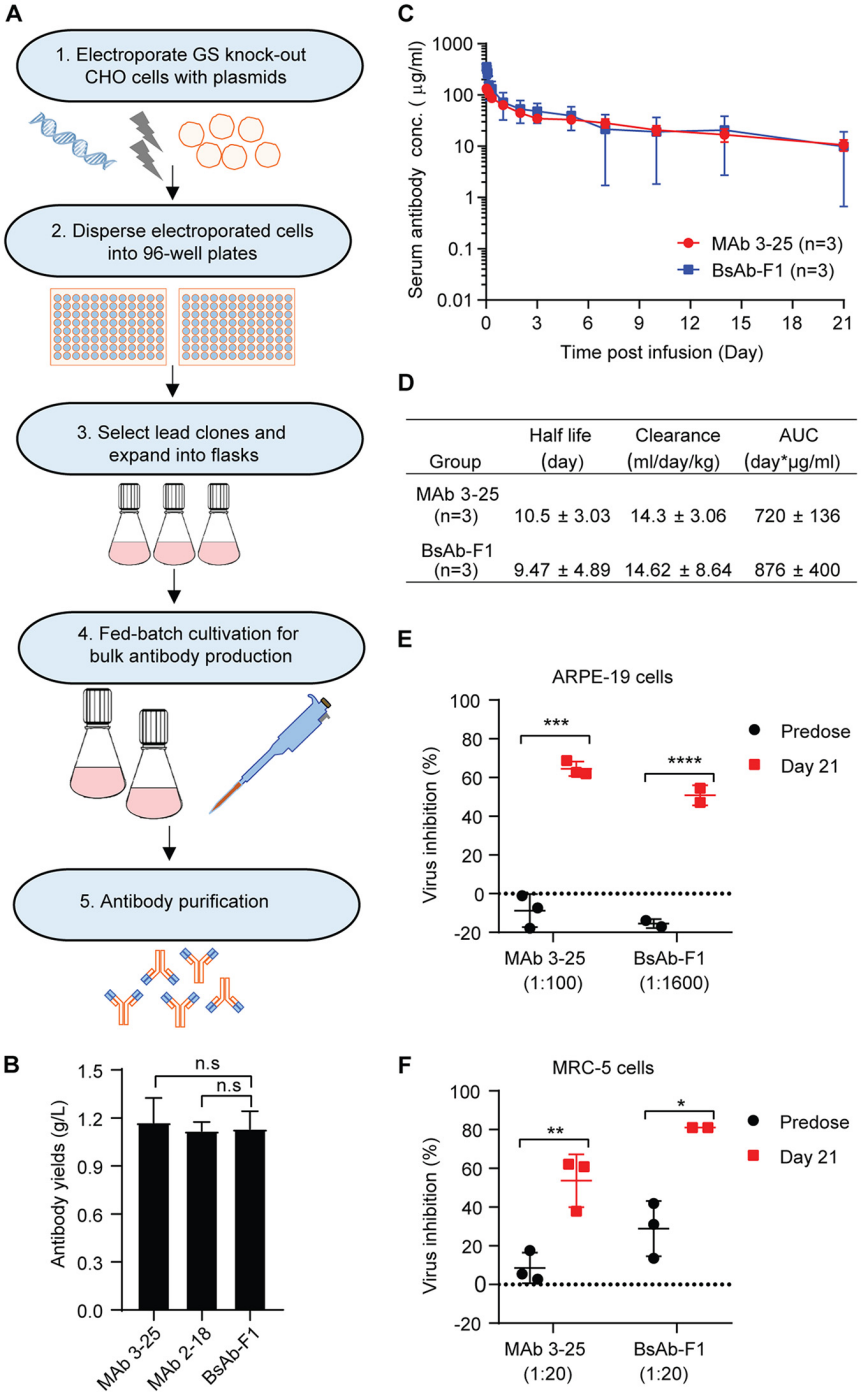
Single-dose pharmacokinetics study of BsAb-F1 and MAb 3-25 in rhesus macaques. (A) A flowchart for construction of stable CHO cell lines. Linearized plasmids with genes for expression of the heavy chain and light chain of the antibody and glutamine synthetase (GS) were transfected into GS knockout CHO cells by electroporation. The transfected cells were dispersed into 96-well plates and cultured under selection medium without glutamine. After 3 weeks, the level of antibody secreted in the culture medium from wells of expanded clones was quantified by ELISA. The high-yield clones were selected for expansion in flasks and used for bulk antibody production by a fed-batch method. (B) The yields of MAb 2-18, MAb 3-25, and BsAb-F1 by lead clones at 16 days postinoculation using fed-batch cultivation. (C) Two groups of rhesus macaques (*n* = 3) were injected intravenously with single dose (10 mg/kg) of MAb 3-25 or BsAb-F1. The serum concentrations of injected antibodies at predose and multiple time points (0.5, 1, 2, 4, and 8 h and 1, 2, 3, 5, 7, 10, 14, and 21 days) postinjection were quantified by ELISA. (D) Pharmacokinetics parameters were calculated by noncompartmental analysis using Phoenix 32 WinNonlin (8.1.0.3530) software (Certara). Data from three animals are shown as mean ± SD. (E and F) The predose and 21-day postinjection serum samples at indicated dilutions were used for HCMV neutralization assays in ARPE-19 cells (E) and MRC-5 cells (F). The unpaired two-tailed Student's *t* test was used to determine statistical significance. n.s., not significant (*P* > 0.05); *, *P* < 0.05; **, *P* < 0.01. The error bars indicate mean ± SD values from three animals in each group.

Our results showed that the bispecific antibodies have dual binding specificities and combined the neutralization activities of MAb 3-25 and MAb 2-18. To determine the developability of the bispecific antibody, we produced the antibodies in CHO cells. The BsAb-F1, which showed a slightly higher binding and neutralization activities than BsAb-F2, was chosen for further evaluation of developability. As shown in Fig. 5A, linearized plasmids containing the genes for expression of the antibody and the glutamine synthetase (GS) selective marker were transfected into GS knockout CHO cells by electroporation. The cells were then subjected to nutrient selection in glutamine-free medium. The high-yield polyclones were selected and expanded into flasks. Fed-batch cultivation of lead polyclones was performed for bulk antibody production. At 16 days postinoculation, the yields of MAb 2-18, MAb 3-25, and BsAb-F1 were all above 1 g/liter in the cell culture supernatant of polyclones (Fig. 5B), which indicates a good potential for further yield increase through screening of high-yield single clones and optimization of culture conditions. Importantly, the antibodies produced by stable CHO cells had a low-endotoxin level (<1 U/mg), enabling pharmacokinetics (PK) study in nonhuman primates.

BsAb-F1 and MAb 3-25 share the same IgG molecule, except that BsAb-F1 has two extra copies of 2-18 scFv at the heavy-chain C terminus. Thus, we investigated the single-dose PK of BsAb-F1 together with MAb 3-25. Two groups (*n* = 3) of rhesus macaques were intravenously dosed with 10 mg/kg of body weight of MAb 3-25 and BsAb-F1, respectively. Blood samples were collected predose and postdose at multiple time points for quantitation of the injected antibodies. As shown in Fig. 5C, the serum concentrations of both MAb 3-25 and BsAb-F1 indicated a biphasic profile with a relatively fast distribution phase followed by a slower elimination phase during the observation period of 21 days. The estimates of half-life, clearance rate, and area under the concentration-time curve (AUC) of BsAb-F1 were comparable to those of MAb 3-25 (Fig. 5D). It should be noted that one monkey had a BsAb-F1 serum concentration of 22.09 μg/ml at day 5, and then BsAb-F1 could not be detected from day 7 onward, which led to larger standard deviations for the data from the BsAb-F1 group. Serum concentrations of MAb 3-15 and BsAb-F1 in other monkeys were all near 10 μg/ml at 21 days postinjection (Fig. 5C). Importantly, the 21-day serum samples from the BsAb-F1 group showed significantly higher HCMV-inhibiting activities than the predose serum samples at dilutions of up to 1:1,600 in ARPE-19 cells and 1:100 in MRC-5 cells. In contrast, the 21-day serum samples from the MAb 3-25 group showed significant HCMV inhibition at a dilution of 1:100 in MRC-5 cells but not in ARPE-19 cells (Fig. 5E and F). These results suggest that the IgG and scFv components of BsAb-F1 retained HCMV neutralizing activities even after 21 days in circulation.

## DISCUSSION

Congenital HCMV infection and HCMV infection in transplant recipients are unneglectable global burdens (5, 38). No HCMV vaccine is currently approved, and the choice of virus-specific treatment is very limited. CytoGam, which contains pooled immunoglobulin derived from adult human plasma with high-titer HCMV antibody, has been successfully used in combination with antiviral chemotherapies for prophylaxis of HCMV diseases in transplant recipients (39–41). Neutralizing MAbs isolated from HCMV-positive healthy donors may represent a better alternative to CytoGam, considering that the MAbs exhibit much higher potency inhibiting HCMV *in vitro* than does the CytoGam (26, 27, 35). Due to the complicated cell entry mechanism and broad cell-type tropism of HCMV infection, single neutralizing antibodies that target one viral glycoprotein lack either potency or broad cell-type coverage. Two antibody combinations, CSJ148 (42) and RG7667 (43), have been developed and tested in clinical trials for prevention of HCMV infection or reactivation in transplant recipients. Bispecific antibodies have been developed as potential treatments against viral infection, including influenza virus, human immunodeficiency virus (HIV), hepatitis B virus (HBV), dengue virus (DENV), Ebola virus, and Zika virus (44–47). We previously characterized a gB AD-2-specific broadly neutralizing MAb, 3-25, and a pentamer-specific potently neutralizing MAb, 2-18 (33, 35). To combine the high potency of the pentamer-specific antibody and broad cell-type coverage of the gB-specific antibody, we developed a bispecific neutralizing antibody based on MAbs 3-25 and 2-18. Recently, our group and another group have reported the development of anti-CD3/anti-gB-based bispecific T-cell engagers (BiTEs) that redirect cytotoxic T cells for killing of HCMV-infected cells (48, 49). While the anti-gB/CD3-bispecific antibodies aim to eradicate virus-infected cells, our bispecific antibody has the potential to neutralize circulating free virus and inhibit cell-to-cell viral spreading.

We previously demonstrated that the bivalency of MAb 3-25 was required for HCMV neutralization (33). In this study, MAb 3-25 but not 2-18 lost neutralization activity in a divalent scFv-Fc format. The binding of 3-25 IgG to gB was about 25-fold higher than that of the divalent 3-25 scFv-Fc. The significantly lower gB binding ability of 3-25 scFv-Fc is probably the major cause of the loss of neutralization activity. Based on the characterization of scFv format of MAbs 3-25 and 2-18, we chose the IgG-scFv format to build the anti-gB- and antipentamer-bispecific neutralizing antibodies. In our design, the 2-18 scFv was connected to the C terminus of MAb 3-25 HC. The original LC of MAb 3-25 was retained. With two identical HCs and LCs, our IgG-scFv bispecific antibody did not have an HC-LC-mispairing issue and preserved the neutralizing activities of both parental antibodies. Small format variations, such as VH/VL orientations in scFv, were shown to significantly impact the expression and antigen-binding activity of a diabody targeting epidermal growth factor receptor (EGFR) and insulin-like growth factor receptor (IGFR) (50). In our case, the bispecific antibodies with two VH/VL orientations in 2-18 scFv did not show a substantial difference in antibody expression, antigen binding, and virus neutralization activities.

Dual specificity is indispensable for bispecific molecules, combining the therapeutic advantages of two individual antiviral antibodies. We used two different assays to measure binding activities: the ELISA, which measures endpoint binding to antigen immobilized on a solid phase, and the BLI assay, which measures dynamic binding to antigen diluted in solution. Both assays confirmed the dual binding specificities of the bispecific antibodies. The binding activities of bispecific antibodies to gB measured by ELISA were consistent with those by the BLI assay. However, there was a discrepancy in the ELISA and BLI assay measurements of binding activities of bispecific antibodies to the pentamer. We speculate that immobilization of the pentamer on a solid phase may cause steric hindrance of binding by the larger IgG-scFv bispecific antibody. In addition, we detected simultaneous binding of bispecific antibodies to the 3-25 epitope peptide and pentamer via ELISA but could not detect the simultaneous binding of bispecific antibodies to gB protein and pentamer, suggesting that binding of one antigen causes steric hindrance for the bispecific antibody binding the other antigen. This partially explains that the IgG-scFv is comparable to but generally not significantly more potent than the most potent parental antibody. However, the dual specificities of IgG-scFv both contribute to virus neutralization and expand the cell-type coverage. The direct cell-to-cell viral spreading is an important strategy of human viruses to evade neutralizing antibodies (51). The direct cell-to-cell transmission of HCMV depends on the pentameric complex and fusion protein gB (20, 37). Our results demonstrate that our bispecific antibodies efficiently neutralized free virus infection of multiple cell lines and also inhibited postinfection viral cell-to-cell spread in APRE-19 cells. Importantly, the performance of bispecific antibodies in HCMV inhibition were comparable to the simple combination of the two parental antibodies. Compared to the combination of the two parental MAbs, the IgG-scFv bispecific antibody is more cost-effective to produce and formulate.

We constructed stable CHO cell lines for production of the bispecific antibody. Without optimization of culture conditions, the polyclonal stable CHO cells were able to produce MAb 3-25 and BsAb-F1 at yields above 1 g/liter using a fed-batch cultivation method. This result suggests a good potential for bioprocess development of this HCMV IgG-scFv bispecific antibody. Results of a single-dose PK study of BsAb-F1 in rhesus macaques indicate that the BsAb-F1 has a PK profile comparable to that of the parental MAb 3-25, which has the same IgG molecule as BsAb-F1. Importantly, the serum samples from the monkeys injected with the BsAb-F1 at 21 days postinjection retained broadly HCMV neutralizing activities in representative cell lines.

Overall, we have developed a fully functional IgG-scFv bispecific neutralizing antibody targeting the HCMV gB and pentamer. To our knowledge, this represents the first IgG-scFv-based bispecific neutralizing antibody against HCMV infection. The bispecific antibody possesses the combined neutralization potency and breadth of the two parental monoclonal antibodies and has the potential to be developed into a therapy for HCMV infection.

## MATERIALS AND METHODS

### Cell lines, virus, and reagents.

MRC-5 (ATCC CCL-171) human embryo lung fibroblasts and HFF-1 (ATCC CRL-1635) human foreskin fibroblasts were cultured in Dulbecco’s modified Eagle’s medium (DMEM) supplemented with 10% fetal bovine serum (FBS). ARPE-19 (ATCC, CRL-230) human retinal pigment epithelial cells were cultured in DMEM–F-12 (50/50) supplemented with 10% FBS. STTG1 (ATCC CRL-3271) neuronal cells were cultured in RPMI 1640 medium supplemented with 10% FBS. Human vascular endothelial cells (HUVECs) were cultured in endothelial cell growth medium (Cell Application, Inc.). Suspension-adapted CHO-S cells were cultured in CD optiCHO medium. GS knockout CHO-S cells were made in-house by CRISPR and cultured in CD OptiCHO medium (catalog no. 10743029; Fisher Scientific) supplemented with 8 mM l-glutamine (catalog no. 35050-061; Life Technologies Corporation). The recombinant gB protein (33) and recombinant pentamer protein (52) were described previously. The HCMV strain AD169 modified for restoration of pentamer expression and insertion of an in-genome GFP expression cassette (named AD169rev-GFP) was described previously (36).

### Construction of plasmids for expression of antibodies.

The gene sequences and plasmids for heavy chains and light chains of MAbs 3-25 and 2-18 were described previously (32). The gene fragments encoding the scFvs of MAbs 2-18 and 3-25 were synthesized and inserted into an expression plasmid to be expressed as a fusion protein with human CH1-Fc. To construct the plasmids for the extra-long heavy chain of IgG-scFv bispecific antibodies, the genes for 2-18 scFv with a (G_4_S)_3_ linker at the N terminus were inserted into the MAb 3-25 heavy-chain expression vector at the end of the gene for the heavy chain. The genes for the heavy chain and light chain of MAb 3-25, MAb 2-18, or bispecific antibodies were inserted into a single plasmid with two expression cassettes and the selective marker glutamine synthetase (GS) for construction of stable CHO cell lines expressing the antibodies.

### Transient expression of scFv-Fc and bispecific antibodies.

The plasmids for scFv-Fc or a 1:1 mixture of heavy-chain and light-chain plasmids for the bispecific antibodies were cotransfected into HEK293 cells in the presence of branched polyethylenimine (PEI) (Sigma, St. Louis, MO). The cell supernatants were harvested at 6 days posttransfection and purified using protein A Sepharose resin. Eluted fractions containing antibodies were immediately neutralized with 1 M Tris-base at pH 8.0 (5% [vol/vol]), concentrated, and buffer exchanged into phosphate-buffered saline (PBS) by ultracentrifugation, quantified by Nanodrop 2000C, validated by SDS-PAGE, aliquoted, and stored at –80°C for later analysis.

### ELISA.

Antigen (gB or pentamer) in phosphate-buffered saline at a concentration of 2 μg/ml was immobilized on Costar 96-well high-binding plates (50 μl/well) at 4°C overnight. The antigen-coated plates were then washed with PBST (PBS with 0.05% Tween 20) three times to remove excess antigens. Plates were blocked with 5% (vol/vol) nonfat milk in PBST (200 μl/well) followed by incubation with serially diluted antibodies (100 μl/well) at 37°C for 1 h. A total of 50 μl/well of horseradish peroxidase (HRP)-conjugated goat anti-human IgG (1/5,000 dilution) was added to the plates and incubated at 37° for 35 min. Washing 5 times with PBST was performed after each incubation step. After the final wash, the plates were developed with TMB (3,3′,5,5′-tetramethylbenzidine) substrate. Absorbance at 450 nm was recorded on a Molecular Devices Spectra Max M4 machine.

### Biolayer interferometry assay.

The binding avidities of parental and bispecific antibodies were assessed on an Octet RED 96 (Fortebio, Menlo Park, CA). Activation of protein A biosensors (Fortebio) was performed by incubation in 1× PBS at room temperature for 30 min. The activated protein A biosensors were loaded with either the parental or bispecific antibodies (20 μg/ml for 180 s). A short baseline step was added (60 s) before and after the loading step. Then, an association step was performed in diluted gB or pentamer antigens at indicated concentrations for 180 s, followed by a dissociation step for 600 s. The sensors were regenerated by dipping into 100 mM glycine buffer (pH 2.6). All samples were diluted in 1× kinetics buffer, with the exception of the gB sample, which was diluted in PBS. One nonloaded biosensor was allowed to associate with antigens to exclude nonspecific binding of antigens on biosensor. One antibody-loaded sensor associated with buffer only served as a reference. The data were analyzed using Octet Data Analysis software version 10.0 (ForteBio). Binding curves were fitted in a 1:1 binding model.

### *In vitro* neutralization assay.

The HCMV neutralization assay was performed as described as previously (33). Briefly, 96-well plates were seeded with the cells 1 day ahead of the experiment, and the cells were allowed to reach ∼95% confluence. HCMV strain AD169rev-GFP was diluted in medium so that 50 μl of diluted virus would produce approximately 200 GFP-positive cells in a specified type of cells after infection. A total of 50 μl of 2-fold-diluted antibody was mixed with an equal volume of diluted virus and incubated at 37°C for 30 min. For the combination of two MAbs, MAbs 3-25 and 2-18 were mixed in a ratio of 1:1. Then the culture medium of cells was removed, and the 100-μl antibody-virus mixture was added to each well. Virus-only controls and noninfected controls were included in each plate. Virus infection was monitored through GFP expression. Due to the different viral susceptibilities of the cells, virus infection was examined at 2 days postinfection in ARPE-19 and MRC-5 cells and at 5 days postinfection in the HFF cells, STTG-1 cells, and HUVECs. Single whole-well GFP expression images were acquired using a CTL Immunospot analyzer. The number of GFP-positive cells in each well was counted automatically using the software of the Immunospot analyzer. The percentage of virus inhibition by the antibody was calculated using the following formula: inhibition % = 100 − (no. of GFP^+^ cells of a sample – no. of GFP^+^ cells of mock-infected cells)/(no. of GFP^+^ cells of virus-only control – no. of GFP^+^ cells of mock-infected cells) × 100. No GFP^+^ cells could be detected in mock-infected cells. The IC_50_ values were calculated by nonlinear fit of virus inhibition percentage versus concentration (nM) using the GraphPad Prism 5 software.

### Inhibition of postinfection viral release and cell-to-cell spreading.

Clear-bottom 96-well plates (Corning Costar) were seeded with ARPE-19 cells (7,000 cells/well) in 100 μl of cell culture medium and incubated overnight at 37°C in a 5% CO_2_ incubator. Cells were then infected with AD169-rev-GFP at about 100 PFU/well in a total volume of 100 μl. At 3 days postinfection, fresh medium with or without 10 μg/ml tested antibodies was used to replace the medium of infected cells. Antibodies were replenished every 72 h. At 14 days postinfection, whole-well images of GFP expression were acquired using a CTL Immunospot analyzer. The number and size of GFP-positive viral plaques were determined using ImageJ software.

### Construction of stable CHO cell lines for bulk antibody production.

GS knockout CHO cells were passaged every 3 days and split 1 day prior to electroporation. The electroporation procedure was described previously (53). Briefly, plasmids with the genes for the antibody HC and LC and the selective GS marker were linearized and then transfected into the host cell line by electroporation using a MaxCyte transfection system. The electroporated cells were then cultured in glutamine-free medium for selection. The lead clones were selected by antibody quantification in the supernatants via ELISA. The antibody was produced from lead clones by inoculation of approximately 0.3 × 10^6^ viable cells per ml into CD FortiCHO medium followed by fed-batch cultivation (37°C, 5% CO_2_, 135 rpm) for 16 days. Nutrient supplement was added to the culture every other day starting at day 3. The density and viability of cells were determined before each feeding. Supernatant harvest and antibody purification followed procedures described previously (54).

### Pharmacokinetics in nonhuman primates.

Rhesus macaques were prescreened for undetectable HCMV neutralization titer and dosed intravenously with 10 mg/kg antibodies (<1 endotoxin unit/mg). Antibody injections and collection of blood samples were performed in the New Iberia Research Center (NIRC). All groups contained 3 subjects, with equal numbers of male and female animals. Serum samples were collected at predose and multiple time points (0.5, 1, 2, 4, and 8 h and 1, 2, 3, 5, 7, 10, 14, and 21 days) postadministration. Serum concentrations of MAb 3-25 and BsAb-F1 were quantified by ELISA. Briefly, 2 μg/ml of gB served as coating antigen. The serum samples at different dilutions were applied to the antigen-coated and 5% nonfat milk-blocked plates and then probed with HRP-conjugated goat anti-human (Fab)_2_ antibody (1:5,000 dilution). Signals were developed with TMB substrate. Absorbance at 450 nm was recorded. Standard curves were generated in the same way using purified MAb 3-25 and BsAb-F1 at a starting concentration of 300 ng/ml and followed by 10 serial 1.5-fold dilutions. The standard curves (optical density at 450 nm [OD_450_] readings versus concentrations) were fitted by a cubic regression formula with *R*^2^ values over 0.99. The OD_450_ reading of the predose sample was subtracted from that of the postinjection samples of the same monkey and then used for calculation of the serum antibody concentration. Pharmacokinetic parameters were calculated by noncompartmental analysis using Phoenix 32 WinNonlin (8.1.0.3530) software (Certara).

### Statistical analysis.

Statistical significance was determined by unpaired two-tailed Student's *t* test using GraphPad Prism 5 software, and is indicated as follows in Fig. 4 and 5: n.s., not significant (*P* > 0.05); *, *P* < 0.05;**, *P* < 0.01; or ***, *P* < 0.001. All results are shown as mean values ± standard deviation (SD) unless otherwise indicated.
